# Surface Effect on Oil Transportation in Nanochannel: a Molecular Dynamics Study

**DOI:** 10.1186/s11671-017-2161-2

**Published:** 2017-06-15

**Authors:** Haixia Zheng, Yonggang Du, Qingzhong Xue, Lei Zhu, Xiaofang Li, Shuangfang Lu, Yakang Jin

**Affiliations:** 1 State Key Laboratory of Heavy Oil Processing, China University of Petroleum, Qingdao, 266580 Shandong People’s Republic of China; 2 College of Science, China University of Petroleum, Qingdao, 266580 Shandong People’s Republic of China; 3 Institute of Unconventional Oil & Gas and New Energy, China University of Petroleum, Qingdao, 266580 Shandong People’s Republic of China

**Keywords:** Nanochannel, Oil transportation, Surface effect, Intermolecular interaction, Momentum transfer

## Abstract

In this work, we investigate the dynamics mechanism of oil transportation in nanochannel using molecular dynamics simulations. It is demonstrated that the interaction between oil molecules and nanochannel has a great effect on the transportation properties of oil in nanochannel. Because of different interactions between oil molecules and channel, the center of mass (COM) displacement of oil in a 6-nm channel is over 30 times larger than that in a 2-nm channel, and the diffusion coefficient of oil molecules at the center of a 6-nm channel is almost two times more than that near the channel surface. Besides, it is found that polarity of oil molecules has the effect on impeding oil transportation, because the electrostatic interaction between polar oil molecules and channel is far larger than that between nonpolar oil molecules and channel. In addition, channel component is found to play an important role in oil transportation in nanochannel, for example, the COM displacement of oil in gold channel is very few due to great interaction between oil and gold substrate. It is also found that nano-sized roughness of channel surface greatly influences the speed and flow pattern of oil. Our findings would contribute to revealing the mechanism of oil transportation in nanochannels and therefore are very important for design of oil extraction in nanochannels.

**Electronic supplementary material:**

The online version of this article (doi:10.1186/s11671-017-2161-2) contains supplementary material, which is available to authorized users.

## Background

Inspired by the ever increasing world energy demand and the excessive consumption of conventional energy, the development of unconventional shale oil has gained extensive attention, because of its large reserves and potential production [1]. Shale oil is short for mature organic shale oil and the most representative energy listed under the unconventional energy headings. Although the world’s total known resources of shale oil is more than three times higher than that of the remaining conventional crude oil [2], the total exploitable reserves of shale oil has been estimated to be much less than the reserves. Besides, oil shale has been exploited for about 200 years, but the development and utilization of shale oil are restricted greatly so far. All these suggest that shale oil, confined in nanochannel, is difficult to be extracted [3]. The channel size of oil shale ranges from 2 to 100 nm in width [4, 5], which generates a large specific surface area and many kinds of surface effect. Under the influence of surface interaction between fluid and substrate, lots of new physical phenomena may be aroused, for example, water flows much faster inside nanotubes than in a classical macroscale tube [6, 7], anomalous increase is found in carbon capacitance at pore sizes less than 1 nm [8], water affinity in carbon nanotube changes from hydrophobic to hydrophilic as the width decreases [9]. Being located in oil shale, with the strong surface interaction between fluids and shale substrate, the fluid exhibits a lot of different characters from that in macroscopic channel, such as the density distribution, wettability, and diffusion coefficient [10–12], resulting in different transportation properties of fluids through such nanochannel from those in macroscale channel. Using molecular dynamics (MD) simulations, Chen et al. investigated the transportation behavior of water inside a model carbon nanotube and found that the shearing stress between the fluid and the channel was size-sensitive, and they also verified the simulative conclusions by experiment on a nanoporous carbon in glycerin [13]. Xue et al. considered flow of decane in silica nanochannel under the driving force from gas flooding, and they found that initial pressure and interaction energy between oil and substrate played an important role in displacement of oil droplets [14]. Wang et al. simulated the flow of octane in quartz slits by MD simulations, and they found that the velocity enhanced with the increase of external force, channel width, and temperature, and they also found that the surface effect can dominate the transportation of oil in the nanochannel with decreasing channel width [15]. As mentioned above, the strong surface interaction between fluids and nanochannel has a crucial effect on the flow of the fluid in nanochannel. However, there is little systematic study on the effect of surface properties on the dynamics mechanism of oil transportation in nanochannel. Understanding the influence of surface effect on the transportation of shale oil in nanochannel is of great significance to promote the development and utilization of shale oil.

In this work, we investigate the dynamics mechanism of oil transportation in nanochannel using MD simulations and demonstrate that the surface interaction between oil molecules and channel surface, roughness of channel surface, and the interaction among oil molecules all have great effects on the center of mass (COM) displacement of oil in nanochannel. The conclusion will not only provide a bright future for the energy field but also shed light on a wide range of natural science, such as environment, biomedicine, chemical, energy, and industrial applications including protein translocation, membrane separation of mixtures, and channel battery [16–20].

## Methods

All MD simulations are carried out by Discover code in the Material Studio (Accelrys Inc.) software. A condensed-phase optimized molecular potential for atomistic simulation studies (COMPASS) is used to describe the interatomic interactions. The COMPASS force field is a general all-atom force field based on ab initio and parameterized using extensive data for molecules in the condensed phase. The force field potential can be expressed as follows:

1 $$ {E}_{\mathrm{total}}={E}_{\mathrm{valence}}+{E}_{\mathrm{cross}-\mathrm{term}}+{E}_{\mathrm{nonbond}} $$

In the above equation, *E*_valence_ refers to valence (or bonding) energy, which is generally accounted for by diagonal terms such as bond stretching, valence angle bending, dihedral angle torsion, and inversion. *E*_cross-term_ refers to cross-terms energy, which accounts for factors such as bond or angle distortions caused by nearby atoms to accurately reproduce the dynamic properties of molecules. And *E*_nonbond_ refers to non-bonding energy, which accounts for the interactions between non-bonded atoms and results mainly from van der Waals (vdW) interactions and electrostatic interactions. The three terms can be represented as

2 $$ \begin{array}{c}{E}_{\mathrm{valence}}={\displaystyle \sum_b\left[{K}_2{\left( b-{b}_0\right)}^2+{K}_3{\left( b-{b}_0\right)}^3+{K}_4{\left( b-{b}_0\right)}^4\right]}\\ {}\kern2.5em +{\displaystyle \sum_{\theta}\left[{H}_2{\left(\theta -{\theta}_0\right)}^2+{H}_3{\left(\theta -{\theta}_0\right)}^3+{H}_4{\left(\theta -{\theta}_0\right)}^4\right]}\\ {}\kern2.5em +{\displaystyle \sum_{\phi}\left[{V}_1\left[1- \cos \left(\phi -{\phi}_1^0\right)\right]+{V}_2\left[1- \cos \left(2\phi -{\phi}_2^0\right)\right]+{V}_3\left[1- \cos \left(3\phi -{\phi}_3^0\right)\right]\right]}\\ {}\kern4.5em +{\displaystyle \sum_{\chi}{K}_{\chi}{\chi}^2+{E}_{\mathrm{UB}}}\end{array} $$

3 $$ \begin{array}{l}{E}_{\mathrm{cross}\hbox{-} \mathrm{term}}={\displaystyle \sum_b{\displaystyle \sum_{b^{\prime }}{F}_{b{ b}^{\prime }}\left( b-{b}_0\right)\left({b}^{\prime }-{b}_0^{\prime}\right)}}\\ {}+{\displaystyle \sum_{\theta}{\displaystyle \sum_{\theta^{\prime }}{F}_{\theta {\theta}^{\prime }}\left(\theta -{\theta}_0\right)\left({\theta}^{\prime }-{\theta}_0^{\prime}\right)}}+{\displaystyle \sum_b{\displaystyle \sum_{\theta}{F}_{b\theta}\left( b-{b}_0\right)\left(\theta -{\theta}_0\right)}}\\ {}+{\displaystyle \sum_b{\displaystyle \sum_{\phi}{F}_{b\phi}\left( b-{b}_0\right)\times }}\left[{V}_1 \cos \phi +{V}_2 \cos 2\phi +{V}_3 \cos 3\phi \right]\\ {}+{\displaystyle \sum_{b^{\prime }}{\displaystyle \sum_{\phi}{F}_{b^{\prime}\phi}\left({b}^{\prime }-{b}_0^{\prime}\right)\left({b}^{\prime }-{b}_0^{\prime}\right)\times }}\left[{F}_1 \cos \phi +{F}_2 \cos 2\phi +{F}_3 \cos 3\phi \right]\\ {}+{\displaystyle \sum_{\theta}{\displaystyle \sum_{\phi}{F}_{\theta \phi}\left(\theta -{\theta}_0\right)\times }}\left[{V}_1 \cos \phi +{V}_2 \cos 2\phi +{V}_3 \cos 3\phi \right]\\ {}+{\displaystyle \sum_{\phi}{\displaystyle \sum_{\theta}{\displaystyle \sum_{\theta^{\prime }}{K}_{\phi \theta {\theta}^{\prime }} \cos \phi \left(\theta -{\theta}_0\right)\times \left({\theta}^{\prime }-{\theta}_0^{\prime}\right)}}}\end{array} $$

4 $$ {E}_{\mathrm{non}\hbox{-} \mathrm{bond}}={\displaystyle \sum_{i> j}\left[\frac{A_{i j}}{r_{i j}^9}-\frac{B_{i j}}{r_{i j}^9}\right]}+{\displaystyle \sum_{i> j}\frac{q_i{q}_j}{\varepsilon {r}_{i j}}}+{E}_{\mathrm{H}\hbox{-} \mathrm{bond}} $$

where *b* and *b*′ are the bond lengths of two adjacent bonds, and *θ*, *ϕ*, and *χ* are the two-bond angle, dihedral torsion angle, and out-of-plane angle, respectively. *q* is the atomic charge, *ε* is the dielectric constant, *r*_*ij*_ is the *i*-*j* atomic separation distance. *b*_0_, *K*_*i*_ (*i* = 2 − 4), *θ*_0_, *H*_*i*_ (*i* = 2 − 4), ${\phi}_i^0$ (*i* = 1 − 3), *V*_*i*_ (*i* = 1 − 3), ${F}_{b{ b}^{\prime }}$, ${b}_0^{\prime }$, ${F}_{\theta {\theta}^{\prime }}$, ${\theta}_0^{\prime }$, *F*_*bθ*_, *F*_*bϕ*_, ${F}_{b^{\prime}\theta}$, *F*_*i*_ (*i* = 1 − 3), *F*_*θϕ*_, ${K}_{\phi \theta {\theta}^{\prime }}$, *A*_*ij*_ and *B*_*ij*_ are fitted from quantum mechanics calculations and are implemented into the Discover module of Materials Studio. Lennard-Jones potential is employed to describe intermolecular interactions between oil molecules, oil molecule and nanochannels [14, 21, 22]. The cutoff distance of 15.5 Å is selected to calculate the vdW interactions, and the Ewald method and the atom-based method are applied for the calculation of electrostatic interactions and vdW interactions, respectively. The system is calculated under constant volume and constant temperature, i.e., the NVT ensemble is employed. The temperature is 298 K, and the Andersen thermostat method is chosen to control the system at a thermodynamic temperature. The periodic boundary condition is imposed in all three dimensions. Data is collected every 5 ps, and the full accurate trajectory is recorded.

**Fig. 1:**
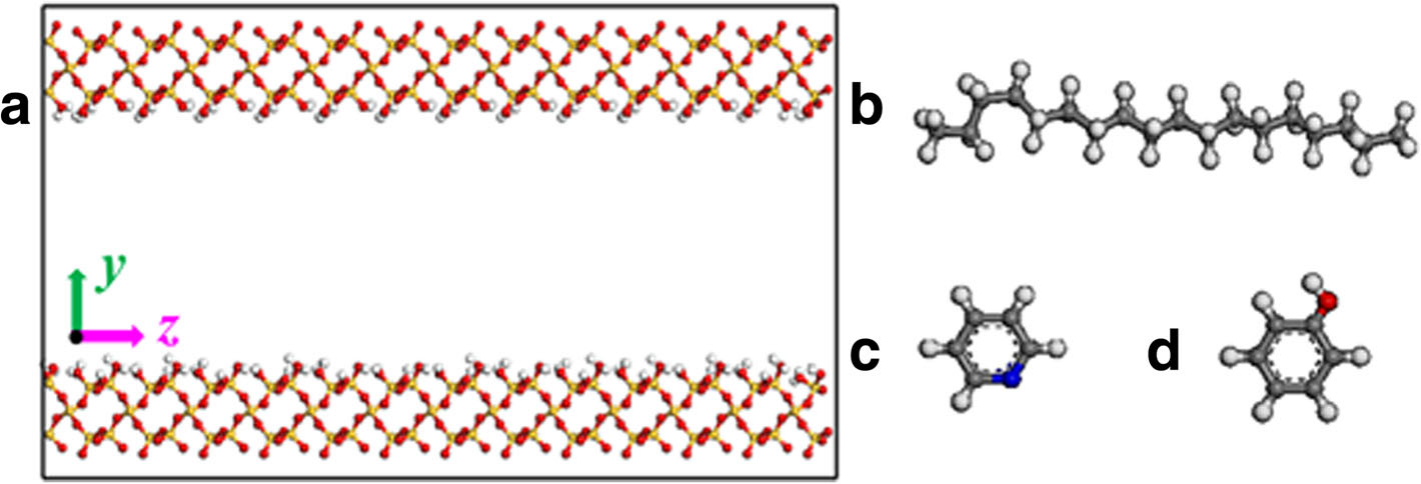
**a** Representation of the silica nanochannel model. Molecular structures of **b** octadecane, **c** pyridine, and **d** phenol. Color code for atoms: *red*, oxygen; *white*, hydrogen; *yellow*, silicon; *grey*, carbon; and *blue*, nitrogen

A major composition of rock minerals is silica in most shale formations [23–25]. So, the silica surface is selected as oil shale surface in our simulation. The initial lattice of silica comes from the database of the Material Studio software. The (0 0 1) surface is cleaved, and then a rectangular surface is refined. The dimensions of each substrate surface are 1.5 × 7 × 0.85 nm^3^. A separation channel along the *z*-axis is created between the two substrate surfaces as shown in Fig. 1a. Channel surfaces are fully modified by hydroxyl to represent geologic conditions [26].

The initial configuration of the system is built by octadecane molecules packing inside the silica channel. Forty octadecane molecules are inserted into the slit channel with a width of 2 nm, leading to a density of 0.8 g/cm^3^. We also study transportation properties of pyridine and phenol molecules, another two components of shale oil, to investigate the effect of oil molecules on oil transportation in nanochannel. The structures of octadecane, pyridine, and phenol are extracted from the database of the Material Studio software, as shown in Fig. 1b–d. To ensure a similar density of oil, the numbers of pyridine molecules, phenol molecules, and octadecane molecules in channels with widths of 4 and 6 nm in our simulations are 407, 344, 80, and 120, respectively.

Using Discover Minimization, we first perform energy minimization to optimize the system so that the system is well equilibrated. Equilibrium simulations are performed using a 500 ps prerun to ensure that the system has reached a steady state. Then non-equilibrium simulations are performed by applying a gravity-like force parallel to channel surface (along the *z*-axis) to all oil molecules in order to promote transportation through channels, which is commonly used in simulation of fluid transportation [27–29]. We note here that one limitation of the MD simulation is that a force comparable to those in ambient settings is impractical, due to the time required for MD calculations; thus, we applied a force that yields an average value of 3.1 × 10^−14^ N on each atom. The intention of the large force is to obtain more precise data for oil transportation given a finite simulation time.

## Results and Discussion

### Effect of Channel Width

**Fig. 2:**
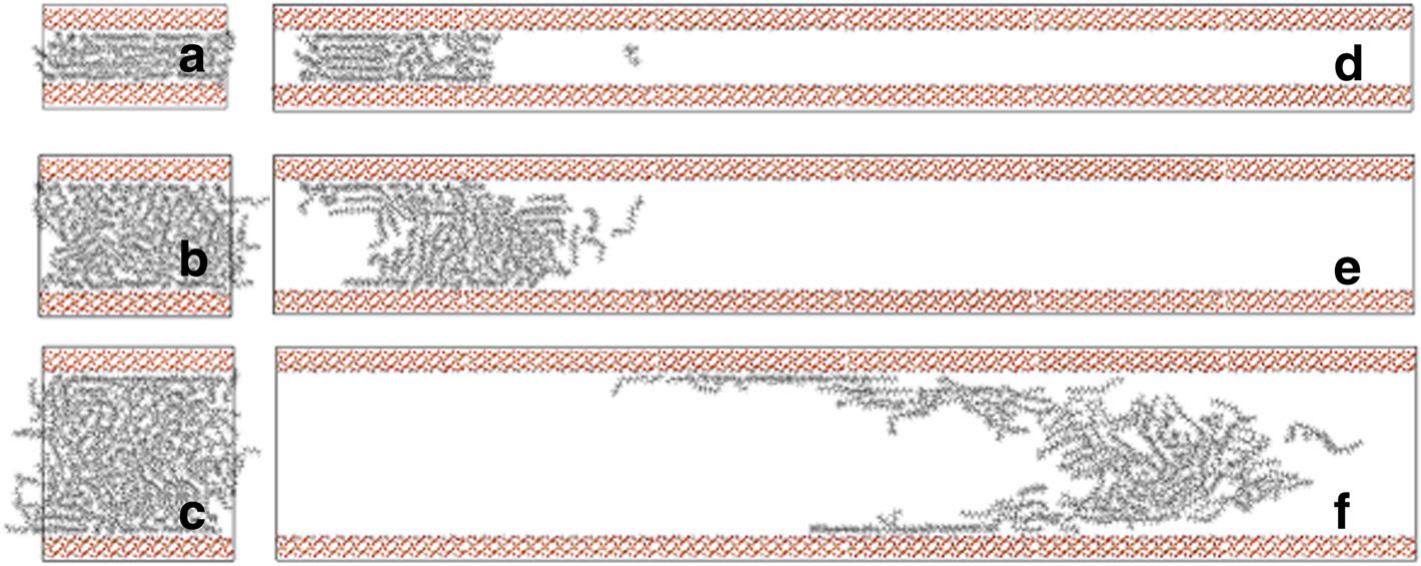
The initial model of force-driven octadecane molecules transportation process in silica channels with widths of **a** 2, **b** 4, and **c** 6 nm, and snapshots of octadecane molecules in **d** 2, **e** 4, and **f** 6 nm channels at 2 ns

We first pay our attention to the effect of channel width on transportation properties of oil. Under the action of the external force, the number of atoms flowing through the cross-section of the channel gradually increases with simulation time (Additional file 1: Figure S1, Supporting Information). The oil molecules are pulled for about 2 ns. As shown in Fig. 2d–f, with the increment of the channel width, the displacement distance of oil is larger after 2 ns MD simulations. To quantitatively describe the oil displacement along the channel axis, we calculate the COM displacement of oil between its initial location and  final location after 2 ns. MD simulations and its initial location along the *z*-axis and the center of mass are defined in terms of a mass-weighted average of the atom coordinates.

5 $$ {z}_{\mathrm{COM}}={\displaystyle \sum_i\frac{m_i}{M}{r}_i} $$

**Fig. 3:**
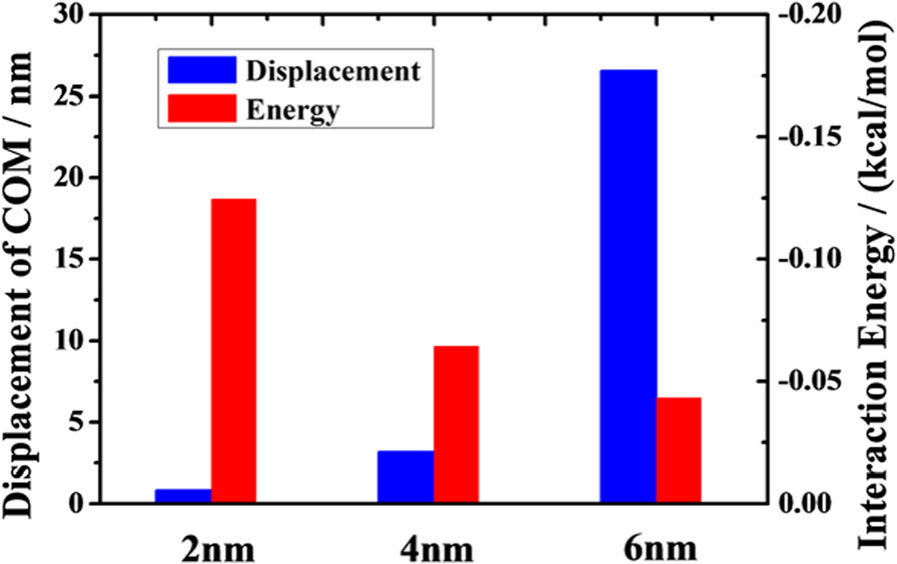
The COM displacements of oil at 2 ns and average interaction energies between the oil and the channel versus channel width

In Fig. 3, we present the oil displacement after 2 ns MD simulations. The results show that under the condition of same pulling force on each atom, the COM displacement of oil in 2 nm channel is only 0.85 nm, which is far smaller than that in 6 nm channel. It suggests that the narrower channel provides a more pronounced adsorption constraint on oil molecules.

To make clear the effect of adsorption constraint, we calculate the average interaction energy between the oil molecules and the substrate. The average interaction energy is calculated as follows:

6 $$ {E}_{\mathrm{average}\ \mathrm{interaction}}=\frac{E_{\mathrm{total}}-\left({E}_{\mathrm{oil}}+{E}_{\mathrm{substrate}}\right)}{N} $$

where *E*_average interaction_ is the average interaction energy between the oil molecule and the substrate; *E*_total_ represents the total energy of the whole system; *E*_oil_ and *E*_substrate_ are the energy of oil components and substrate components, respectively; and *N* is the total number of atoms of oil molecules [14, 30, 31]. Figure 3 displays that the oil displacement decreases with the increase of average interaction energy. It is obvious that the adsorption between oil molecules and channel grows with interaction energy. The strong adsorption of channel inhibits the oil transportation in narrow channel. From the data illustrated in Fig. 3, we find that when the interaction energy is enhanced three times, the oil displacement decreases by more than 30 times. It suggests that the transportation of oil is greatly influenced by the interaction between oil molecule and substrate. However, this effect decreases with increasing channel width. The size effect on oil transportation is more obvious in nanochannel than in microchannel (Additional file 1: Figure S2). Therefore, reducing the interaction energy between oil molecule and substrate is a key factor for enhancing the oil transportation in nanochannel.

**Fig. 4:**
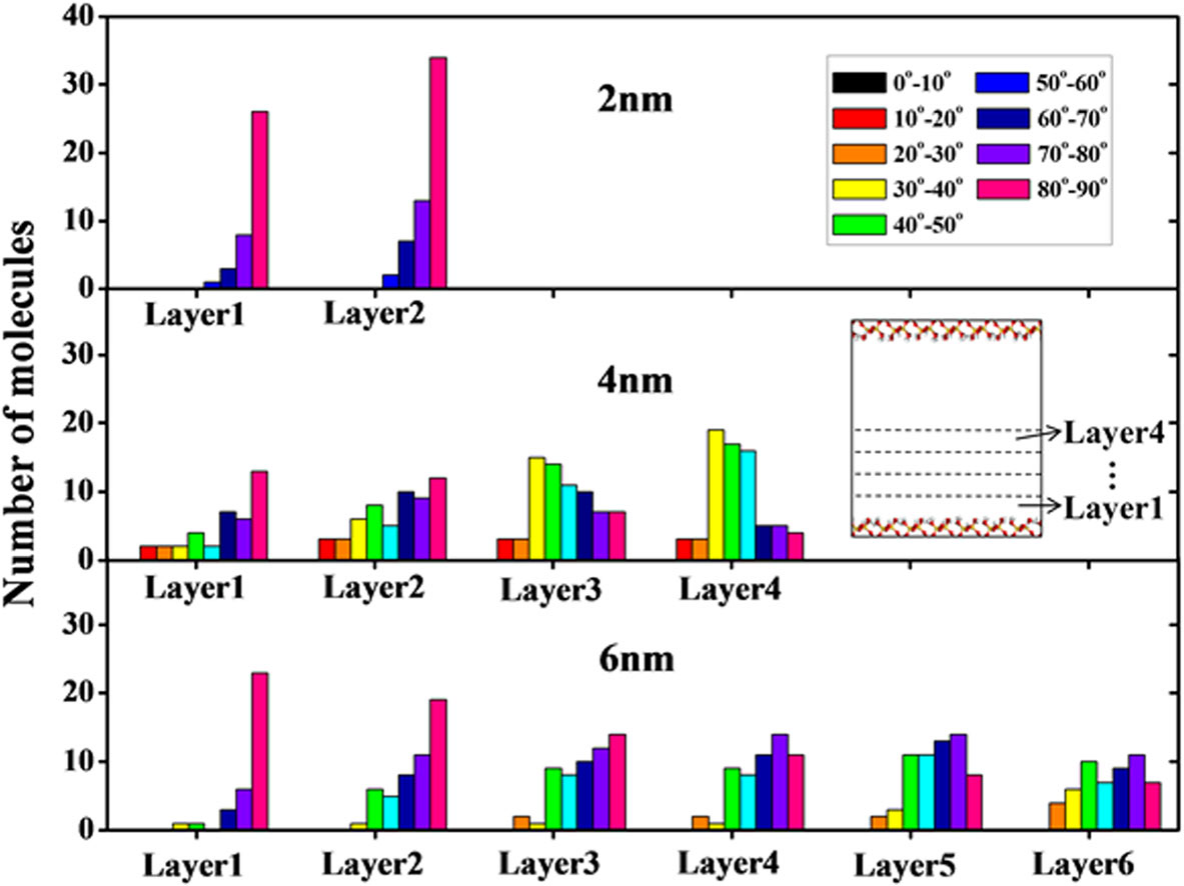
The orientation angle distribution of octadecane molecules in each layer for different channel width

**Fig. 5:**
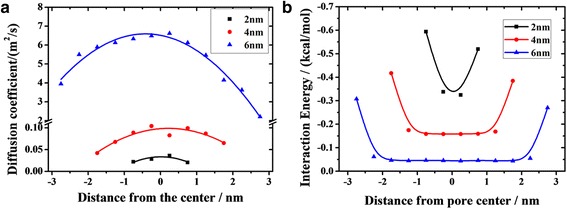
**a** Diffusion coefficient profiles of oil in channels with different width. **b** Distance dependence of average interaction energy between oil molecules and silica (with symbols). Solid lines represent the fitting functions

It can be seen from Fig. 2 that there have been obvious layering structures near the nanochannel surface, with a thickness of approximately 5 Å. It should be noted that the layer contacting the channel surface and the layer in the center of channel are called contact layer and central layer, respectively. Obviously, well-ordered oil molecule layers are found in the near-surface region. The orientation of the octadecane molecules is commonly characterized by the angle *θ* between the normal vector to the channel surface and some vectors which are formed by the line connecting the two carbon atoms at the end of an octadecane molecule [15, 29]. The orientation distribution for octadecane molecules in each layer at 2 ns is presented in Fig. 4. Here, *θ* = 80^o^ ∼ 90^o^ corresponds to parallel orientation of the molecule, whereas a value of *θ* = 0^o^ ∼ 10^o^ means that the molecule is perpendicular to the channel surface. One can see that the octadecane molecules are mainly parallel to the surface in layers of 2 nm channel and in the contact layer of 4 nm channel and 6 nm channel, due to the strong oil-surface interaction (Fig. 5b). For the central layers of 4 nm channel and 6 nm channel, there is no preferential orientation of octadecane molecules, which means that those octadecane molecules tend to lie at various angles to the channel surface. The aligned octadecane molecules in the contact layer can be important for transport properties of oil molecules in nanochannel.

Next, we observe that the startup times (defined as the time at which the displacement of a layer is more than 5 Å) for various layers are different by checking the trajectories. The startup time data listed in the supporting information (Additional file 1: Table S1) shows that the startup time of contact layer increase with decreasing channel width, which means that pulling force needed to start the moving of a contact layer in a narrower channel is larger than that required in a wider channel. Besides, the startup time of central layer is much earlier than that of the contact layer.

In addition, we find that the flow rate of oil decreases with increasing distance from the channel axis, and the flow rate of the contact layer decreases with decreasing channel width (Fig. 2d–f). To quantitatively describe these characters, we study the diffusion coefficient of oil molecules at a different location away from the channel center, which is obtained from the time evolution of the mean-square-displacement according to

7 $$ D=\frac{1}{4}\underset{t\to \infty }{ \lim}\frac{\mathrm{d}}{\mathrm{d} t}\left\langle {\left|{r}_i(t)-{r}_i(0)\right|}^2\right\rangle $$

where *r*_*i*_ denotes the position vector of *i*th particle, and the angular brackets denote an ensemble average. Figure 5a shows how the diffusion coefficients of layers depend on the position in nanochannel. The curves of 4 nm channel and 6 nm channel present a parabola style, i.e., toward the channel surface, the diffusion coefficients of layers decrease gradually. The 6-nm channel shows the largest difference between the high value and low value of 4.4 m^2^/s, while the 2-nm channel holds the minimum difference of 0.016 m^2^/s. The diffusion coefficients of layers in 2-nm channel are slightly different, so the front surface of oil looks like a piston. Besides, we find that the diffusion coefficients of the layers at the same distance from the channel surface are quite different for various channels (Fig. 5a). For example, the diffusion coefficient of the layer contacting the lower channel surface in 6 nm channel is 3.9 m^2^/s, while that in 2 nm channel is only 0.02 m^2^/s. It means that the flow rate of layers at the same distance from the channel surface increases with increasing channel width.

**Fig. 6:**
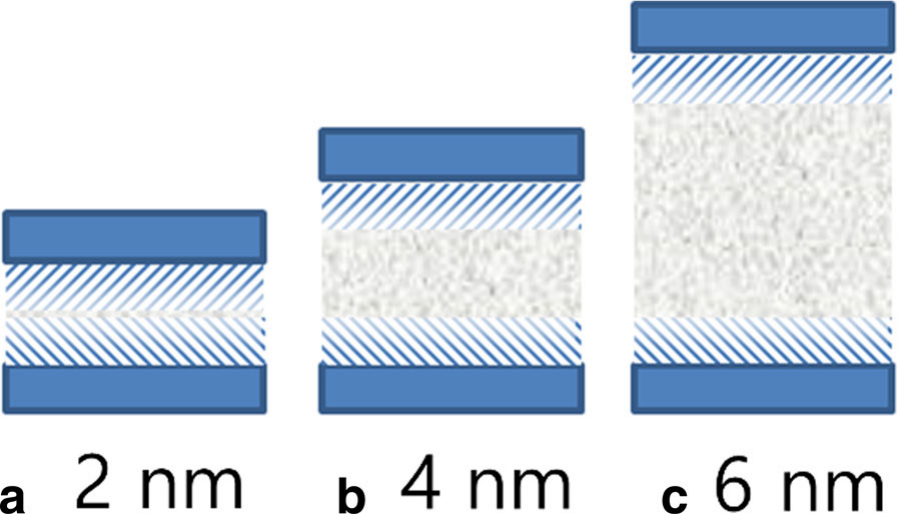
**a**, **b**, **c** Schematic of the range of adsorption between oil and substrate

In Fig. 5b, we present the average interaction energy between the oil molecules at different location away from the channel center and the channel. The interaction energies are obviously larger at both ends of the curves and decrease rapidly within 1 nm as the substrate has strong adsorption on oil molecules in the range of 1 nm, and it is one of the reasons for the slow start of contact layers. However, the interaction energies between the oil molecules and the channel cannot adequately explain the shape of the front surface, for its values for layers outside of the range of strong adsorption are nearly same. The parabola front surface is not only related to the interaction between the oil and the channel but also to the interaction among oil molecules. Intermolecular interaction contributes to the fluid viscosity, which plays an important role in momentum transfer in viscoelastic fluid. Since the range of the adsorption of channel on oil is about 1 nm, some layers are located in the area, as are marked by shadow in Fig. 6. The strong surficial interactions between oil and substrate block the transportation of oil layers in shadow. Momentum is transferred from layers out of the shadow to the layers in the shadow. The number of atoms out of the shadow decreases with decreasing channel width. Thereby, less momentum is transferred to the layers in shadow in narrower channel. So, the diffusion velocity of contact layer decreases with decreasing channel width.

### Effect of Polarity

**Fig. 7:**
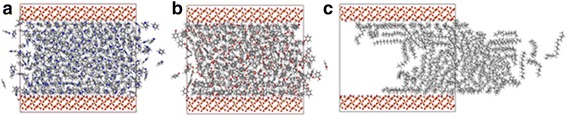
Snapshots of **a** pyridine, **b** phenol, and **c** octadecane transportation in 4 nm silica channels at 2 ns

**Fig. 8:**
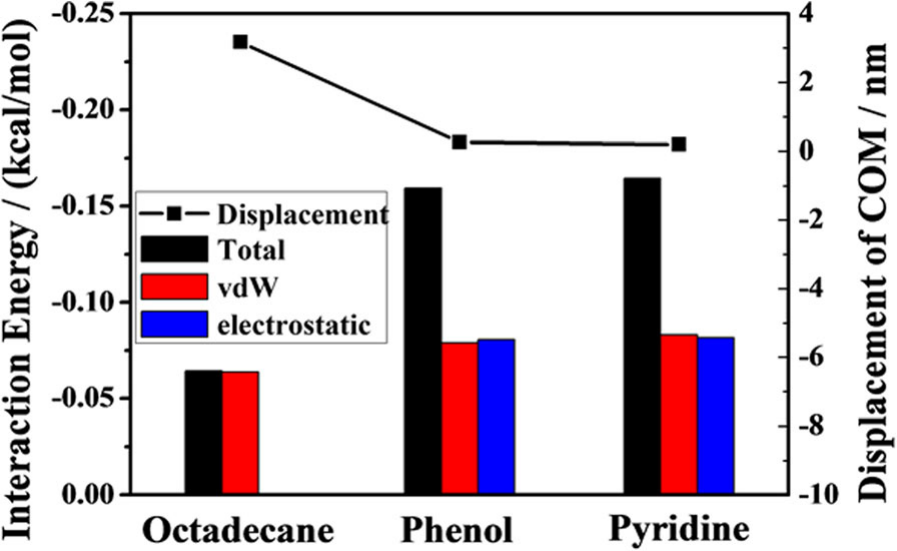
The COM displacements of oil at 2 ns and average interaction energies (total, vdW, and electrostatic) between the different components and the channel

Shale oil always contains the components of polar oil, and these polar oil components play an important role in the adsorption of the oil/silica interface [21, 32], so understanding their impact on oil transportation is crucially important. With phenol and pyridine for example, we perform simulations in a 4-nm channel, and the atom numbers of phenol and pyridine are approximately equal to the atom number of octadecane in 4-nm channel. The snapshots of pyridine, phenol, and octadecane in the silica channel at 2 ns are shown in Fig. 7. Compared with the octadecane molecules, phenol molecules and pyridine molecules nearly cannot be driven by pulling force. The broken line in Fig. 8 shows the COM displacements of different oil molecules after 2 ns MD simulations. Although the pulling forces on each atom are equal, the COM displacement of octadecane is almost 16 times as large as the COM displacement of phenol and pyridine.

In order to understand these results, we calculate the energy of the total interaction, vdW interaction, and electrostatic interaction between the different components and the channel. The histogram in Fig. 8 illustrates that the total interaction energy between phenol (pyridine) and silica substrate is larger than that between octadecane and silica channel. Because octadecane molecule is a chain, non-polar molecule, the total interaction between the octadecane molecules and the channel results mainly from vdW interaction, and there is little electrostatic interaction, while the contributions of vdW interaction and electrostatic interaction between phenol (pyridine) and the channel to the total interaction are nearly same.

To investigate the influence of polarity on oil transport, we calculate the dipole momentum of the three molecules using the first-principle simulation. The details of performance follow our previous work [33–36]. Results show that dipole momentums of octadecane, phenol, and pyridine are 0.0322, 1.3059, and 2.2449 Debye, respectively. It indicates that the polar oil molecules are much more difficult to be driven than the nonpolar molecules in nanochannel. But the COM displacement of oil does not always increase with decreasing polarity. For the two polar oil molecules, the polarity of phenol is weaker than the polarity of pyridine, but the COM displacements of them are almost equal.

### Effect of Types of Materials

**Fig. 9:**
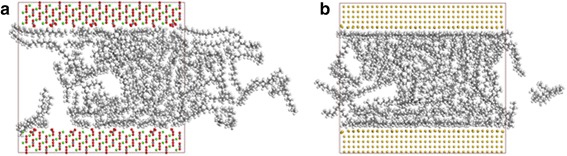
Snapshots of octadecane transportation in 4 nm **a** calcite channel and **b** gold channel at 2 ns. Color code for atoms: green, calcium; yellow, gold

The transportation characters of oil molecules are also compared among those nanochannels fabricated from various types of materials, including silica, gold, and calcite. Figure 9 shows the snapshots of octadecane molecules in calcite and gold channels at 2 ns. Figure 9a shows a distinct transportation of oil molecules in calcite channel, indicating that the octadecane molecules in calcite can also be driven by the pulling force, while the molecules in gold channel can hardly move (Fig. 9b).

**Fig. 10:**
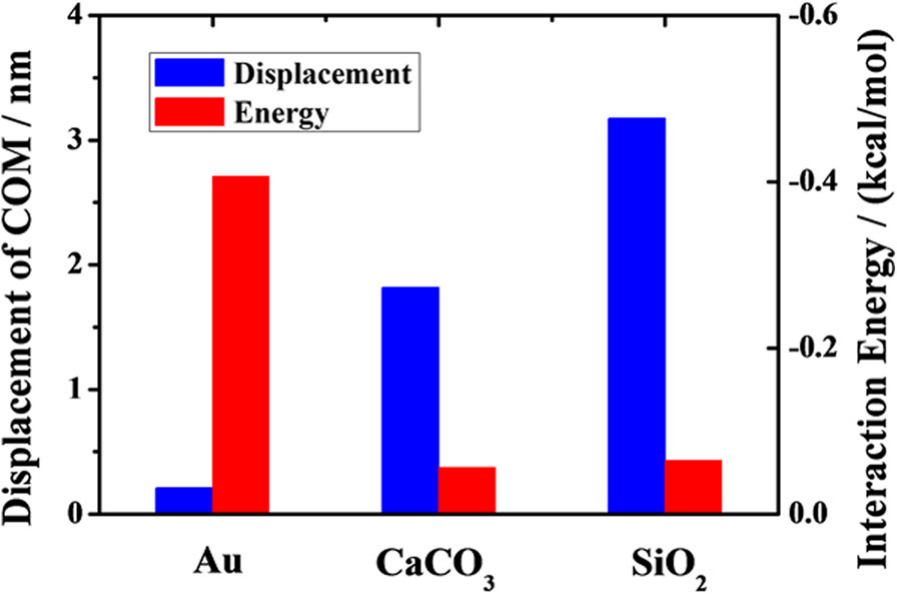
The COM displacements of oil at 2 ns and average interaction energies between oil and substrates of different materials

Figure 10 shows the COM displacements of octadecane molecules in channels of various materials and the average interaction energies between oil and different material channels. The COM displacement of oil in silica channel is much larger than that in gold channel. The phenomenon can be explained by the effect of the interaction between the oil molecules and the channel. The average interaction is much smaller between the oil molecules and the silica channel than between the oil molecules and the gold channel. But for the transportation of the oil molecules in the silica channel and in the calcite channel, this factor cannot adequately explain the difference. The average interaction energy between the oil molecules and the silica channel does not appear to be much different from that between the oil molecules and the calcite channel, but the COM displacements in the two cases are quite different. The reason may be correlated to the surface atoms and the surface texture. These results indicate that the oil transportation is greatly influenced by the interaction between oil molecules and channel, but when the values of the interaction energy are similar, the oil transportation in nanochannel is the competition among those factors.

### Effect of Surface Roughness

**Fig. 11:**
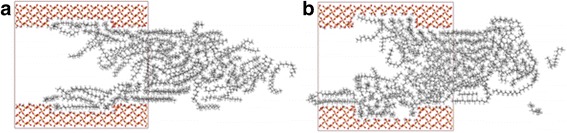
Snapshots of octadecane transportation in rough channel with the cavity depth of **a** 3 and **b** 6 Å at 2 ns

As well known, nano-sized surface roughness has little influence on the fluid flows inside micro-sized channels. However, it has been demonstrated that the nano-sized surface roughness has great influences on fluid transportation in nanochannels [37–39]. To investigate the effect of roughness on transportation of octadecane, we construct rough surfaces by cutting out a small amount of atoms from the substrate surface, so that a cavity with a depth of *d* = 3 Å (or 6 Å) and a width of 35 Å is formed on substrate surface. The naked oxygen atoms were modified by hydrogen atoms. Five and ten octadecane molecules are added to 3 Å cavity and 6 Å cavity, respectively, and the external force is increased correspondingly. Figure 11 shows the comparison of snapshots for octadecane flowing through rough channel with cavity depths of 3 and 6 Å at 2 ns. We observe that inside every cavity, there are some oil molecules, and their localizations are affected by the cavity, which results in a reduction of velocity values inside the cavity, as well as the velocity of oil molecules nearby. And this becomes more obvious when *d* = 6 Å, as shown in Fig. 11b. To quantify the influence of roughness on transportation, we further calculate the COM displacement of oil in rough channels. The COM displacements of oil in channels with 3 and 6 Å depth cavity are 3.95 and 3.07 Å, respectively. When *d* = 6 Å, the value of oil displacement is 3.07 Å, which is smaller than the value 3.17 Å of oil molecules in flat channel. Somewhat surprisingly, however, for *d* = 3 Å, the displacement is even larger than that in flat channel. We expect that these characters are contributed by two parts: (1) the cavity increases the width of the nanochannel so that the oil molecules have a greater diffusion coefficient according to the above discussion, which facilitates the transportation of oil; (2) the oil molecules in cavity can suppress the transportation of oil molecules nearby and therefore decrease the oil transportation speed. For the oil molecules in channel with *d* = 3 Å, the effect of suppression caused by the less oil molecules in cavity is less than the effect of facilitation caused by the width increment. When *d* = 6 Å, the diffusion coefficient of oil molecules is further increased; however, more oil molecules are suppressed by the deeper cavity, and the effect of suppression on the transportation of oil molecules is more than that of facilitation, thereby reducing the oil displacement. Because of these complications, we cannot separate these parts and judge how much contribution of each part has on the displacement.

## Conclusions

In this study, we investigate the mechanism of oil transportation in nanochannels using molecular dynamics simulations. It is demonstrated that the oil displacement in a 6 nm channel is over 30 times larger than that in a 2 nm channel, and the diffusion coefficient of oil molecules at the center of the 6 nm channel is almost two times more than that near the channel surface, due to interaction difference between the oil molecules and channels. Besides, we find that both the polarity of oil molecules and channel component have great effects on the interaction between oil molecules and channel in the channels with same width; the larger the interaction between oil molecules and channel is, the smaller the oil displacement is. Finally, we demonstrate that surface roughness can obviously affect oil transportation in nanochannels. The mechanism by which the cavity structure affects the transportation of oil is an intricate issue, which should be further studied. Our findings would contribute to revealing the mechanism of oil transportation in nanochannels and therefore are very important for design of oil extraction in nanochannels.

## Additional file

Additional file 1: Supporting Information for Surface Effect on Oil Transportation in Nanochannel: A Molecular Dynamics Study. (DOCX 225 kb)

## Abbreviations

COM: Center of mass
COMPASS: Condensed-phase optimized molecular potential for atomistic simulation studies
MD: Molecular dynamics
vdW: van der Waals
