# Supplementary material for: Surface Effect on Oil Transportation in Nanochannel: a Molecular Dynamics Study
Source: Nanoscale Res Lett. 2017 Jun 15;12:413. doi: 10.1186/s11671-017-2161-2 (PMC5472647; doi:10.1186/s11671-017-2161-2)
Supplement: Additional file 1: — Supporting Information for Surface Effect on Oil Transportation in Nanochannel: A Molecular Dynamics Study. (DOCX 225 kb) [file 11671_2017_2161_MOESM1_ESM.docx]

Additional file

# Surface Effect on Oil Transportation in Nanochannel： A Molecular Dynamics Study

Haixia Zheng^1,2^, Yonggang Du^2*^, Qingzhong Xue^1,2*^,

Lei Zhu^2^, Xiaofang Li^2^, Shuangfang Lu^3^, and Yakang Jin^2^

* Correspondence

^1^ State Key Laboratory of Heavy Oil Processing, China University of Petroleum, Qingdao 266580, Shandong, P. R. China

^2^ College of Science, China University of Petroleum, Qingdao 266580, Shandong, P. R. China

^3^ Institute of Unconventional Oil & Gas and New Energy, China University of Petroleum, Qingdao 266580, Shandong, P. R. China

Haixia Zheng **E-mail:** [zhenghx@upc.edu.cn](mailto:zhenghx@upc.edu.cn)

Yonggang Du **E-mail:** [duyg@upc.edu.cn](mailto:duyg@upc.edu.cn)

Qingzhong Xue **E-mail:** [xueqingzhong@tsinghua.org.cn](mailto:xueqingzhong@tsinghua.org.cn)

Lei Zhu **E-Mail:** [zhuleiupc@126.com](mailto:zhuleiupc@126.com)

Xiaofang Li **E-mail:** [lixf19901314@163.com](mailto:lixf19901314@163.com)

Shuangfang Lu **E-mail:** [lushuangfang@upc.edu.cn](mailto:lushuangfang@upc.edu.cn)

Yakang Jin **E-mail:** jinyakang9209@163.com

**Fig. S1** Number of extracted atoms versus simulation time (with symbols). Solid lines represent the fitting functions.

The dynamic process of the oil transport in nanochannel can be examined in a quantitative fashion by counting the number of atoms through the cross section of the channel with increasing widths against the simulation time, as shown in Fig. S1. It shows that the number of atoms increases gradually with the simulation time during the transport process.

**Fig. S2** Relative change in flow rate obtained from MD simulation *v*_MD_ (black dots) and the average flow rate estimated from Poiseuille equation *v*_P_ (red dots) against the channel width

The size effect on oil transport in nanochannel is investigated by comparing the flow rate of COM measured from MD simulations and that estimated from Poiseuille equation for micrometer sized pores, namely

$v=\frac{Q}{S}=\frac{\pi\Delta pR^{4}}{8\eta lS}$ (1)

where *Q* is the total flux and *S* is the cross sectional area. The viscosity 1cp (which is assumed not to vary spatially), channel length 7 nm, pressure difference , are substituted for $\eta$, $l$ and $\Delta p$ in eq. 1, and the pulling force *F* is consistent with the value used in MD simulation. Because the flux of atom changes slowly with pulling time(Fig. S1 of the Supporting Information), the flow rate *v*_MD_ can be obtained by dividing the COM displacement by the pulling time 2 ns. The relative change in flow rate is calculated using the following formula

$\Delta_{i=2,4}=\frac{v_{6}-v_{i}}{v_{6}}\times100$ (2)

Fig. S2 shows that, when the channel width is reduced to 4 nm and 2 nm, the reduction of *v*_MD_ is larger than that of *v*_P_, which indicates that Poiseuille’s Law underestimates the effect of channel size on oil transport in nanochannel.

**Table S1** The startup times of central layer and contact layer

| Channel width | 2 nm | 4 nm | 6 nm |
| --- | --- | --- | --- |
| Startup time (central layer) | 624 ps | 100 ps | 27 ps |
| Startup time (contact layer) | 1178 ps | 275 ps | 141 ps |
